# Supplementary material for: Acid-base transporters and pH dynamics in human breast carcinomas predict proliferative activity, metastasis, and survival
Source: eLife. 2021 Jul 5;10:e68447. doi: 10.7554/eLife.68447 (PMC8282339; doi:10.7554/eLife.68447)
Supplement: Figure 9—source data 1. — When adjusted for expression of ESR1, PGR, and ERBB2, the mRNA expression of SLC9A1 does not correlate with that of SLC4A7, SLC16A1, and SLC16A3 (n=409–1162). [file elife-68447-fig9-data1.docx]

| Variable *vs.* *SLC9A1* | Pearson correlation coefficient | *P*-value | Control variables | Partial correlation coefficient | *P*-value |
| --- | --- | --- | --- | --- | --- |
| *ESR1* | 0.292 | <0.001 | *PGR, ERBB2* | 0.290 | <0.001 |
| *PGR* | 0.097 | <0.001 | *ESR1*, *ERBB2* | –0.022 | 0.45 |
| *ERBB2* | 0.162 | <0.001 | *ESR1*, *PGR* | 0.187 | <0.001 |
| *SLC4A7* | 0.087 | 0.003 | *ESR1*, *PGR*, *ERBB2* | 0.023 | 0.43 |
| *SLC16A1* | –0.115 | <0.001 | *ESR1*, *PGR*, *ERBB2* | 0.011 | 0.72 |
| *SLC16A3* | –0.068 | 0.17 | *ESR1*, *PGR*, *ERBB2* | 0.021 | 0.68 |
