# Supplementary material for: Acid-base transporters and pH dynamics in human breast carcinomas predict proliferative activity, metastasis, and survival
Source: eLife. 2021 Jul 5;10:e68447. doi: 10.7554/eLife.68447 (PMC8282339; doi:10.7554/eLife.68447)
Supplement: Figure 10—figure supplement 1—source data 1. — When adjusted for expression of ESR1, PGR, and ERBB2, the mRNA expression of SLC16A1 does not correlate with that of SLC4A7, SLC9A1, and SLC16A3 (n=409–1457). [file elife-68447-fig10-figsupp1-data1.docx]

| Variable *vs.* *SLC16A1* | Pearson correlation coefficient | *P*-value | Control variables | Partial correlation coefficient | *P*-value |
| --- | --- | --- | --- | --- | --- |
| *ESR1* | –0.283 | <0.001 | *PGR, ERBB2* | –0.252 | <0.001 |
| *PGR* | –0.177 | <0.001 | *ESR1*, *ERBB2* | –0.078 | 0.008 |
| *ERBB2* | –0.221 | <0.001 | *ESR1*, *PGR* | –0.251 | <0.001 |
| *SLC4A7* | –0.103 | <0.001 | *ESR1*, *PGR*, *ERBB2* | –0.037 | 0.20 |
| *SLC9A1* | –0.115 | <0.001 | *ESR1*, *PGR*, *ERBB2* | 0.011 | 0.72 |
| *SLC16A3* | 0.131 | 0.008 | *ESR1*, *PGR*, *ERBB2* | 0.034 | 0.50 |
