# Supplementary material for: Acid-base transporters and pH dynamics in human breast carcinomas predict proliferative activity, metastasis, and survival
Source: eLife. 2021 Jul 5;10:e68447. doi: 10.7554/eLife.68447 (PMC8282339; doi:10.7554/eLife.68447)
Supplement: Transparent reporting form [file elife-68447-transrepform.pdf]

Westbrook Centre
Milton Road
Cambridge CB4 1YG, UK
P 01223 855340
W elifesciences.org
T @elife

eLife's transparent reporting form

We encourage authors to provide detailed information *within their submission* to facilitate the interpretation and replication of experiments. Authors can upload supporting documentation to indicate the use of appropriate reporting guidelines for health-related research (see EQUATOR Network), life science research (see the BioSharing Information Resource), or the ARRIVE guidelines for reporting work involving animal research. Where applicable, authors should refer to any relevant reporting standards documents in this form.

If you have any questions, please consult our Journal Policies and/or contact us: editorial@elifesciences.org.

Sample-size estimation

- You should state whether an appropriate sample size was computed when the study was being designed
- You should state the statistical method of sample size computation and any required assumptions
- If no explicit power analysis was used, you should describe how you decided what sample (replicate) size (number) to use

Please outline where this information can be found within the submission (e.g., sections or figure legends), or explain why this information doesn't apply to your submission:

No explicit power analysis was performed. All patients within the project period, who fulfilled the criteria, were included in the study. Group sizes were further dependent on the proportion of patients presenting with the respective clinicopathological characteristics. The groups used for statistical comparisons included between 6 and 80 patients, which according to our previous experience (e.g., *Int J Cancer* 2013;132:1288-99, *Pflugers Arch* 2015;467:367-77, *Oncogene* 2016;35:2112-22) provides sufficient statistical strength to identify biologically relevant molecular contributions to net acid extrusion capacity and pH regulation. This is explained in the "Statistics" paragraph of the Materials and Methods section.

Replicates

- You should report how often each experiment was performed
- You should include a definition of biological versus technical replication
- The data obtained should be provided and sufficient information should be provided to indicate the number of independent biological and/or technical replicates
- If you encountered any outliers, you should describe how these were handled
- Criteria for exclusion/inclusion of data should be clearly stated
- High-throughput sequence data should be uploaded before submission, with a private link for reviewers provided (these are available from both GEO and ArrayExpress)

Please outline where this information can be found within the submission (e.g., sections or figure legends), or explain why this information doesn't apply to your submission:

eLife Sciences Publications, Ltd is a limited liability non-profit non-stock corporation incorporated in the State of Delaware, USA, with company number 5030732, and is registered in the UK with company number FC030576 and branch number BR015634 at the address Westbrook Centre, Milton Road, Cambridge CB4 1YG.

The n-values represent biological replicates and each experiment was performed one time under each experimental condition as described in the "Statistics" paragraph of the Materials and Methods Section.
The data are provided in the figures stratified by clinicopathological characteristics. All experiments that produced sufficient intracellular acidification to compute net acid extrusion capacity in the selected pH intervals were included - no outlier values were excluded. The criteria are described in the "Intracellular pH measurements" paragraph of the Materials and Methods section.

eLife Sciences Publications, Ltd is a limited liability non-profit non-stock corporation incorporated in the State of Delaware, USA, with company number 5030732, and is registered in the UK with company number FC030576 and branch number BR015634 at the address Westbrook Centre, Milton Road, Cambridge CB4 1YG.

Statistical reporting

- Statistical analysis methods should be described and justified
- Raw data should be presented in figures whenever informative to do so (typically when N per group is less than 10)
- For each experiment, you should identify the statistical tests used, exact values of N, definitions of center, methods of multiple test correction, and dispersion and precision measures (e.g., mean, median, SD, SEM, confidence intervals; and, for the major substantive results, a measure of effect size (e.g., Pearson's r, Cohen's d)
- Report exact p-values wherever possible alongside the summary statistics and 95% confidence intervals. These should be reported for all key questions and not only when the p-value is less than 0.05.

Please outline where this information can be found within the submission (e.g., sections or figure legends), or explain why this information doesn't apply to your submission:

> The applied statistical tests and n-values are described in the figure legends. Additional information regarding summary statistics and choice of tests is provided in the "Statistics" paragraph of the Materials and Methods section.
> As the groups are typically large, summary data are provided for easier interpretation. Whenever possible, absolute p-values are given (see figures).

(For large datasets, or papers with a very large number of statistical tests, you may upload a single table file with tests, Ns, etc., with reference to sections in the manuscript.)

Group allocation

- Indicate how samples were allocated into experimental groups (in the case of clinical studies, please specify allocation to treatment method); if randomization was used, please also state if restricted randomization was applied
- Indicate if masking was used during group allocation, data collection and/or data analysis

Please outline where this information can be found within the submission (e.g., sections or figure legends), or explain why this information doesn't apply to your submission:

> Patients were allocated to individual groups based on clinicopathological characteristics from the standard diagnostic procedures. This information was not known to the investigators until after the experiments were performed and had been analysed. This information is provided in the "Statistics" paragraph of the Materials and Methods section.

Additional data files ("source data")

- We encourage you to upload relevant additional data files, such as numerical data that are represented as a graph in a figure, or as a summary table
- Where provided, these should be in the most useful format, and they can be uploaded as "Source data" files linked to a main figure or table
- Include model definition files including the full list of parameters used
- Include code used for data analysis (e.g., R, MatLab)
- Avoid stating that data files are "available upon request"

Please indicate the figures or tables for which source data files have been provided:

eLife Sciences Publications, Ltd is a limited liability non-profit non-stock corporation incorporated in the State of Delaware, USA, with company number 5030732, and is registered in the UK with company number FC030576 and branch number BR015634 at the address Westbrook Centre, Milton Road, Cambridge CB4 1YG.

The Figure Supplements provide enhanced details and in-depth presentation of the data suited especially for the more specialist reader. In these figures, net acid extrusion is plotted as functions of intracellular pH rather than summarized for just one pH level. We include de-identified dataset files, where restricted information is organized in intervals of no less than 5 individuals to comply with the requirements of the ethical approval, following consultation with the Regional Committee on Health Research Ethics.

eLife Sciences Publications, Ltd is a limited liability non-profit non-stock corporation incorporated in the State of Delaware, USA, with company number 5030732, and is registered in the UK with company number FC030576 and branch number BR015634 at the address Westbrook Centre, Milton Road, Cambridge CB4 1YG.
